# Association Between Statin Use and Psoriasis in Patients with Dyslipidemia: A Korean National Health Screening Cohort Study

**DOI:** 10.3390/jcm14010091

**Published:** 2024-12-27

**Authors:** Kyeong Min Han, Mi Jung Kwon, Hyo Geun Choi, Ji Hee Kim, Joo-Hee Kim, Dae Myoung Yoo, Na-Eun Lee, Ho Suk Kang

**Affiliations:** 1 Hallym Data Science Laboratory, Hallym University College of Medicine, Anyang 14068, Republic of Korea; km.han@hallym.ac.kr (K.M.H.); ydm@hallym.ac.kr (D.M.Y.); d23009@hallym.ac.kr (N.-E.L.); 2 Department of Pathology, Hallym University Sacred Heart Hospital, Hallym University College of Medicine, Anyang 14068, Republic of Korea; mulank99@hallym.or.kr; 3 Suseo Seoul E.N.T. Clinic and MD Analytics, 10, Bamgogae-ro 1-gil, Gangnam-gu, Seoul 06349, Republic of Korea; mdanalytics@naver.com; 4 Department of Neurosurgery, Hallym University Sacred Heart Hospital, Hallym University College of Medicine, Anyang 14068, Republic of Korea; kimjihee@hallym.or.kr; 5 Division of Pulmonary, Allergy, and Critical Care Medicine, Department of Internal Medicine, Hallym University Sacred Heart Hospital, Hallym University College of Medicine, Anyang 14068, Republic of Korea; luxjhee@hallym.or.kr; 6 Division of Gastroenterology, Department of Internal Medicine, Hallym University Sacred Heart Hospital, Hallym University College of Medicine, Anyang 14068, Republic of Korea

**Keywords:** statin, psoriasis, national health screening cohort, nested case–control study

## Abstract

**Background/Objectives:** Psoriasis is a chronic inflammatory disease that significantly impacts physical and emotional health. Statins, primarily used as lipid-lowering drugs, have also demonstrated anti-inflammatory effects. While some studies suggest that statins may improve psoriasis symptoms, the findings have been inconsistent. This study aims to investigate the association between prior statin use and the onset of psoriasis in a Korean population, focusing on individuals with dyslipidemia to minimize confounding factors. **Methods**: Using the Korean Health Insurance database (2002–2019), a nationwide nested case-control study was conducted, enrolling 8285 participants with psoriasis and 33,140 controls, matched 1:4 for sex, age, residence, and income through propensity scoring. **Results**: Adjusted odds ratios revealed significantly lower risks of psoriasis among short-term statin users (OR = 0.70, 95% CI = 0.66–0.74) and long-term users (OR = 0.77, 95% CI = 0.73–0.82) than in nonusers. This trend was consistent for both lipophilic and hydrophilic statins, and across subgroup analyses. **Conclusions**: These findings suggest that statins may reduce the incidence of psoriasis. However, further research is needed to assess their effects on psoriasis progression and severity.

## 1. Introduction

Psoriasis is a chronic inflammatory disease with no definitive cure that significantly affects both physical and emotional health and is associated with a low quality of life for patients [1]. While psoriasis is prevalent worldwide, its incidence is higher among Caucasians compared to Asians, resulting in a limited number of large-scale epidemiological studies focused on Asian populations. Previous studies have reported a psoriasis prevalence of 2.5% among Caucasian Americans. It ranges from 0.5% to 1.5% in India, 0.29% to 1.18% in Japan, 0.2% to 1.5% in China, and 0.44% to 0.45% in Korea, indicating a lower prevalence in Asian populations [2,3]. Psoriasis affects the skin and nails and is associated with comorbid conditions, including diabetes, cardiovascular disease, chronic kidney disease, and psoriatic arthritis [4]. Previously regarded as a simple skin disorder, psoriasis is now recognized as one of the most common immune-mediated diseases [5]. It imposes a significant economic burden on patients and healthcare systems and is closely linked to lipid metabolism. One study suggested that lipid-lowering drugs may improve psoriasis skin lesions [6,7,8].

Statins have been widely used as lipid-lowering drugs to reduce cardiovascular disease risk for several decades, as they were the first commercially available drugs of their kind [9]. Additionally, statins have been shown to influence the immune system, reduce inflammation, regulate nitric oxide synthesis, and impact other physiological processes [10,11]. They may also provide potential protective effects against neurological disorders such as Parkinson’s disease, stroke, and multiple sclerosis [12,13,14,15]. Numerous studies have investigated the anti-inflammatory effects of statins, which extend beyond their cholesterol-lowering properties [10]. Statin use has been demonstrated to reduce C-reactive protein (CRP), a clinical marker of inflammation, in patients with various conditions, including chronic kidney disease, type 2 diabetes, and coronary artery disease [16,17,18].

We hypothesized that statins might also effectively treat chronic inflammatory diseases, such as psoriasis, if they exert anti-inflammatory effects. Some studies have suggested that statins can improve the Psoriasis Area and Severity Index (PASI) or reduce the risk of psoriasis [19,20]. However, conflicting results exist, with some studies suggesting that statins may exacerbate psoriasis [21,22]. For example, an Israeli cohort study found that the risk of developing psoriasis varied with the duration of statin use [23]. Despite these findings, large-scale cohort studies focusing on Asian populations are lacking. Statins are classified into two types based on their lipid solubility—lipophilic and hydrophilic statins [24]. While most studies have demonstrated an association between lipophilic statins and psoriasis, the preventative effects of hydrophilic statins remain less well understood [25].

In this study, we utilized a nationwide population-based cohort of Korean adults to test the hypothesis that prior lipophilic and hydrophilic statin use could influence the onset of psoriasis. Given the established association between dyslipidemia and psoriasis [26], we limited our study population to individuals with a history of dyslipidemia or blood cholesterol levels of 200 mg/dL or higher. This approach aimed to reduce the confounding effect of comparing healthy individuals without dyslipidemia to those with the condition, thereby enhancing the accuracy of our analysis.

## 2. Materials and Methods

### 2.1. Ethics

We obtained approval for this analysis from the Hallym University Ethics Committee (IRB No: 2022-12-007; approval date: 15 December 2022). The requirement for written informed consent was waived by the Institutional Review Board. All analyses were conducted in accordance with the guidelines and regulations of the Hallym University Ethics Committee.

### 2.2. Study Design and Participant Selection

This nested case–control study utilized the Korean National Health Insurance Service-Health Screening Cohort (NHIS-HEALS), which includes participants from 2002–2003 health screening programs conducted by the NHIS in the Republic of Korea [27]. The database includes a simple random sample of 1% of the entire population, containing approximately 510,000 participants aged 40–79 years.

Psoriasis participants were selected from among 514,866 participants with 895,300,177 medical claim codes between 2002 and 2019 (*n* = 13,190). Among those with psoriasis, we excluded patients who were diagnosed with psoriasis from 2002–2003 as the washout period (*n* = 2070). Psoriasis patients who had no history of dyslipidemia or total cholesterol <200 mg/dL (*n* = 2832) were excluded. We also excluded patients whose BMI (*n* = 1) or total cholesterol (*n* = 2) data were not available. The control group included all participants, excluding psoriasis participants. Those in the control group who were diagnosed with psoriasis at once were removed (*n* = 11,016).

**Figure 1:**
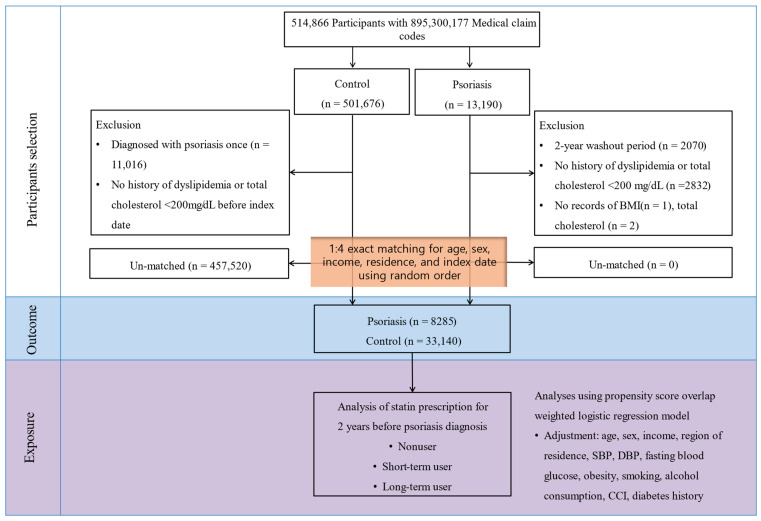
Schematic illustration of the participant selection process in the present study. Among a total of 514,866 participants, 8285 psoriasis participants were matched with 33,140 control participants for age, sex, income, and region of residence.

Age, sex, income, and region of residence were matched between participants in the case and control groups. The index date of psoriasis was defined as the date of the first diagnosis of psoriasis. Similarly, the index date for the control group was set as the date of the matched psoriasis participant. During the matching process, individuals in the control group with no history of dyslipidemia or total cholesterol less than 200 mg/dL were excluded. After matching, 457,520 control individuals were excluded. A final group of 8285 psoriasis patients was paired with a control group of 33,140 participants (Figure 1).

### 2.3. Statin Use (Independent Variable)

The use of statins was extracted from the NHIS database via prescription codes. On the basis of studies indicating that the effects of statins can last for approximately two years, we calculated the cumulative days of statin use by summing the duration of statin use in the two years preceding the diagnosis of psoriasis [28]. Depending on the number of days used within these two years, participants were categorized as nonusers (no statin use), short-term users (1–365 days), or long-term users (more than 365 days). Statins are classified as hydrophilic or lipophilic on the basis of their solubility in water or lipids. In this study, pravastatin and rosuvastatin were categorized as hydrophilic statins, whereas atorvastatin, fluvastatin, lovastatin, pitavastatin, and simvastatin were categorized as lipophilic statins [29].

### 2.4. Psoriasis (Dependent Variable)

Psoriasis was defined via the following ICD-10 codes: L40.0 (psoriasis vulgaris), L40.1 (generalized pustular psoriasis), L40.2 (acrodermatitis continua), L40.4 (guttate psoriasis), L40.5 (arthropathic psoriasis), L40.8 (other psoriasis), L40.9 (psoriasis, unspecified), M07.0 (distal interphalangeal psoriatic arthropathy), M07.1 (arthritis mutilans), M07.2 (psoriatic spondylitis), M07.3 (psoriatic arthropathies) and M09.0 (juvenile arthritis in psoriasis). Participants who visited clinics 2 or more times were selected [30].

### 2.5. Covariates

The age groups were divided into 10 categories at 5-year intervals. Income levels were classified into five categories, from the lowest (1) to the highest (5), and the area of residence was categorized into urban and rural. Obesity was assessed via BMI (kg/m^2^) as follows: <18.5 (underweight), ≥18.5 to <23 (normal), ≥23 to <25 (overweight), ≥25 to <30 (obese I), and ≥30 (obese II). Smoking and alcohol consumption were investigated through self-report questionnaires, while the values for systolic blood pressure (SBP), diastolic blood pressure (DBP), fasting blood glucose, and total cholesterol levels were obtained from health screening exams. The Charlson Comorbidity Index (CCI) was calculated using 17 potential comorbid conditions, with scores ranging from 0 to 29, excluding diabetes [31]. Diabetes history was considered a potential confounding factor if it was diagnosed more than twice with the ICD-10 codes E10–E14.

### 2.6. Statistical Analyses

Propensity score (PS) overlap weights were used to balance the characteristics between the psoriasis group and the control group, enhancing the precision of the analysis. Propensity scores were calculated via multivariate logistic regression with all covariates. When the overlap weights were calculated, a probability of 1-PS was assigned to the psoriasis group, and a probability of PS was assigned to the control group [32]. Standardized differences were calculated to compare the covariate differences between the psoriasis patients and the control group.

To analyse the association between statin use and psoriasis, we utilized a propensity score overlap weighted multivariate logistic regression to calculate odds ratios with 95% confidence intervals. The analysis included a crude model (unadjusted) and an overlap weighted model (adjusted for age, sex, income, region of residence, SBP, DBP, fasting blood glucose, obesity, smoking, alcohol consumption, diabetes history, and CCI scores). In addition, we analysed the associations between the use of any statin and psoriasis, as well as between the use of hydrophilic and lipophilic statins separately. Subgroup analyses were conducted for all covariates. SAS version 9.4 software (SAS Institute, Cary, NC, USA) was used for analysis. All the statistical analyses were two-sided, and *p* < 0.05 was considered significant.

## 3. Results

The psoriasis and control groups were matched on the basis of age, sex, income, and region of residence, resulting in a standardized difference (SD) of 0 for these variables. However, for the other variables that were not matched, the SDs ranged from 0.01 to 0.15. After applying overlap weighting to account for these variables, the standardized differences for all covariates included in the analysis were reduced to 0 between the psoriasis and control groups (Table 1).

Compared with nonusers, short-term users and long-term users had lower odds of psoriasis, with adjusted ORs of 0.70 (95% CI = 0.66–0.74, *p* < 0.001) and 0.77 (95% CI = 0.73–0.82, *p* < 0.001), respectively. Short-term users had lower odds than long-term users did. This trend was also observed for lipophilic statins (OR = 0.70, 95% CI = 0.66–0.74 for short-term users; OR = 0.81, 95% CI = 0.76–0.86 for long-term users) and hydrophilic statins (OR = 0.78, 95% CI = 0.71–0.86 for short-term users; OR = 0.84, 95% CI = 0.75–0.94 for long-term users), and all the results were statistically significant (Table 2).

**Figure 2:**
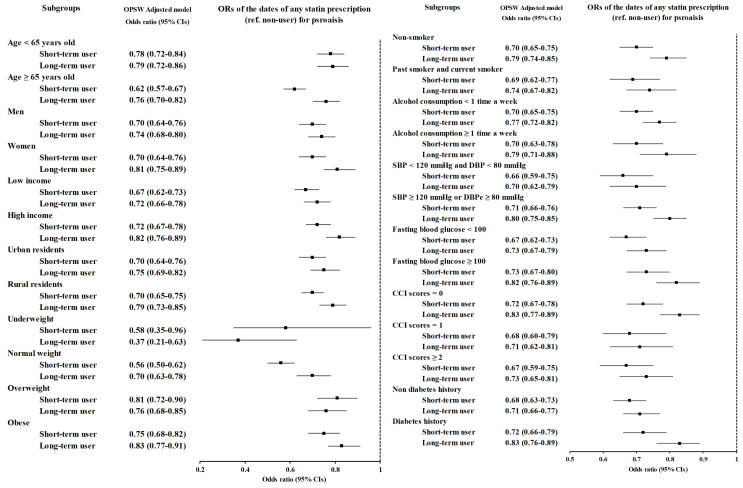
Subgroup analyses of any statin for psoriasis stratified by age, sex, income, region of residence, obesity, smoking status, alcohol consumption, blood pressure, fasting blood glucose, CCI score, and diabetes history.

Subgroup analyses were performed according to age, sex, income, region of residence, obesity, smoking status, alcohol consumption, blood pressure, fasting blood glucose, CCI score, and diabetes history. Consequently, short-term users and long-term users had lower odds than statin nonusers did. Among the two groups, long-term users generally had higher odds. However, short-term users had higher odds than long-term users did in the underweight (OR = 0.58, 95% CI = 0.35–0.96 for short-term users; OR = 0.37, 95% CI = 0.21–0.63 for long-term users) and overweight (OR = 0.81, 95% CI = 0.72–0.90 for short-term users; OR = 0.76, 95% CI = 0.68–0.85 for long-term users) groups. The odds ratio of any statin for psoriasis was the lowest in the normal weight group (OR = 0.56, 95% CI = 0.50–0.62 for short-term users; OR = 0.74, 95% CI = 0.65–0.84 for long-term users). In the overweight group (OR = 0.81, 95% CI = 0.72–0.90 for short-term users; OR = 0.76, 95% CI = 0.68–0.85 for long-term users), the ORs were the highest. (Figure 2 and Table S1).

**Figure 3:**
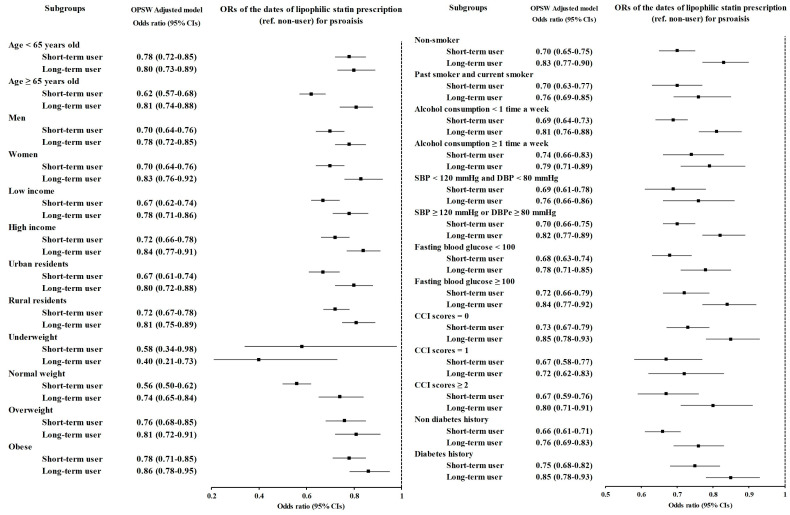
Subgroup analyses of lipophilic statins for psoriasis patients stratified by age, sex, income, region of residence, obesity status, smoking status, alcohol consumption status, blood pressure, fasting blood glucose level, CCI score, and diabetes history.

**Figure 4:**
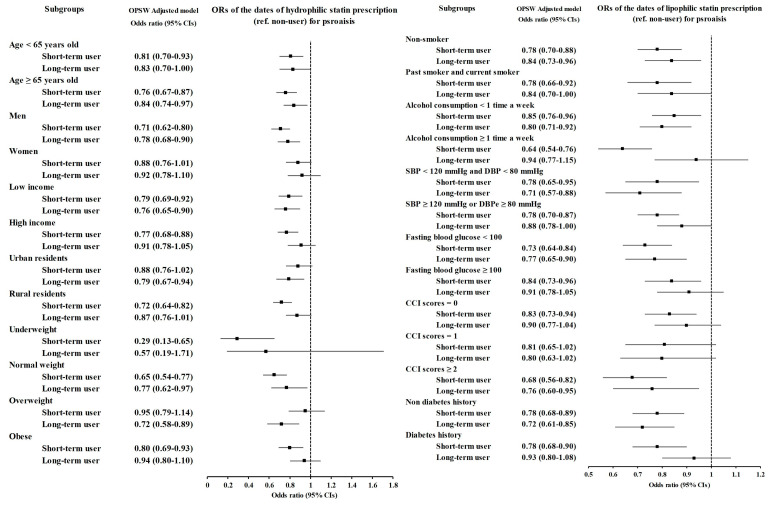
Subgroup analyses of hydrophilic statins for psoriasis patients stratified by age, sex, income, region of residence, obesity status, smoking status, alcohol consumption status, blood pressure, fasting blood glucose level, CCI score, and diabetes history.

Subgroup analyses for both lipophilic and hydrophilic statins also revealed that short-term users had lower odds than long-term users did, similar to the results for any statin. For lipophilic statins, long-term users had lower odds than short-term users did in the underweight group (OR = 0.58, 95% CI = 0.34–0.98, *p* = 0.044 for short-term users; OR = 0.40, 95% CI = 0.21–0.73, *p* = 0.003 for long-term users). For hydrophilic statins, long-term users had lower odds than short-term users in the overweight group (OR = 0.95, 95% CI = 0.79–1.14, *p* = 0.594 for short-term users; OR = 0.72, 95% CI = 0.58–0.89, *p* = 0.003 for long-term users), but this difference was not statistically significant. Short-term users had significantly lower odds of being underweight (OR = 0.29, 95% CI = 0.13–0.65, *p* = 0.002 for short-term users; OR = 0.57, 95% CI = 0.17–1.71, *p* = 0.318 for long-term users) (Figure 3 and Figure 4 and Tables S2 and S3).

## 4. Discussion

In a nationwide cohort study conducted in Korea, we found that statin use is associated with a reduced incidence of psoriasis. Short-term statin use (1 to 365 days) was slightly more effective than long-term use (365 days or more), however, both durations significantly reduced the incidence of psoriasis (OR = 0.70, 95% CI = 0.66–0.74; OR = 0.77, 95% CI = 0.73–0.82, respectively). This result remained consistent across various subgroups, including gender, age, income, region SBP, DBP, fasting blood glucose, obesity, smoking, alcohol consumption, diabetes history, and CCI scores. By comparing patients with dyslipidemia or total cholesterol levels of 200 mg/dL or higher to a matched control group, our study provided a more accurate evaluation of the impact of statin use on psoriasis incidence among individuals with similar health conditions.

A meta-analysis of randomized controlled trials found that statin treatment significantly reduced PASI scores (mean difference = −1.96) at week 8 compared with placebo, although no significant difference was observed after week 12 [33]. However, this meta-analysis included only seven studies with a total of 369 patients, and there was variation in statin use and baseline psoriasis severity among the studies. Large cohort studies on the association between statin use duration and psoriasis risk are limited. An Israeli large cohort study measured statin use by the proportion of days covered (PDC) and reported that patients with a PDC of 40–59% had a significantly reduced risk of psoriasis compared to those with a PDC of <20% (men, HR = 0.84; women, HR = 0.74). In contrast, the risk reduction was significant only in women with a PDC of ≥80% (HR = 0.88) [23]. Similarly, a UK cohort study reported that short-term use (1–4 prescriptions) reduced the risk of psoriasis (OR = 0.60), while long-term use (5–19 or ≥20 prescriptions) did not show a similar effect [34]. These studies lacked proper control groups [23] or matching protocols [34]. In comparison, our study minimized bias by matching the experimental and control groups 1:4 based on age, sex, income, residence, and index date.

The prevalence of metabolic syndrome in patients with psoriasis is estimated to be 20–50%, depending on the severity of the condition [35]. This association may be attributed to overproduction of inflammatory cytokines such as IL-12, IL-23, and TNF-α, which affect systemic metabolism and induce metabolic disorders like insulin resistance [36,37,38]. Psoriasis is significantly associated with type 2 diabetes and obesity (relative risk = 1.18, odds ratio = 1.70, respectively) [39,40]. Globally, 25% of patients with psoriasis are obese, with patients with moderate psoriasis exhibiting a higher prevalence of obesity (36%) compared to those with mild psoriasis (27%) [41]. Major risk factors for metabolic syndrome, such as smoking and alcohol consumption, are also closely associated with psoriasis severity [42,43,44]. Therefore, diabetes, obesity, smoking, and alcohol consumption are important confounding factors when evaluating the relationship between psoriasis and statin use. Previous large-scale epidemiological studies did not adjust for these factors [23,34]. In our study, we used overlap propensity score weighting to balance the characteristics of psoriasis and control groups, effectively reducing the standardized differences for covariates such as diabetes, obesity, smoking, and alcohol consumption to zero (Table 1). Additionally, patients with psoriasis often exhibit lipid metabolism abnormalities, with elevated plasma total cholesterol levels compared to healthy individuals. Normal cholesterol levels are known to lower the risk of developing psoriasis [45,46]. Unlike the cohort studies above [23,34], we limited our population to individuals with total cholesterol levels ≥200 mg/dL or a history of dyslipidemia, further addressing potential biases. By fully utilizing the NHIS database, we aimed to control potential biases and showed that statin use was associated with a reduced risk of psoriasis over short- and long- term durations. Our findings suggest that statin use may have a greater effect on psoriasis development than metabolic syndrome-related factors such as diabetes, obesity, smoking, and alcohol consumption.

The pathogenesis of psoriasis is not fully understood, although it is thought to involve excessive feed-forward activation of the adaptive immune system, leading to hyperproliferation of keratinocytes and accompanying inflammation. Activated myeloid dendritic cells secrete excessive amounts of IL-12 and IL-23. IL-12 differentiates naive T cells into T-helper 1 (Th1) cells and promotes TNFα secretion, while IL-23 induces IL-17 and IL-22 secretion from Th17 and Th22 cells. These secreted cytokines activate intracellular signaling in keratinocytes, leading to hyperproliferation and inflammation [47]. As statins became known to improve the prognosis of patients with heart transplant and those with glomerulonephritis, studies on their immunomodulatory effects began [11]. Statin-induced inhibition of HMG-CoA reductase within the mevalonate pathway reduces levels of farnesyl pyrophosphate (FPP) and geranylgeranyl pyrophosphate (GGPP), key components in the post-translational isoprenylation of cell signaling proteins. A reduction in mevalonate pathway products decreases TNFα synthesis and affects the production of coenzyme Q, an essential substrate of the mitochondrial membrane, essential for mitochondrial energy production [11]. Additionally, statins can dose-dependently inhibit dendritic cell maturation and antigen presentation processes [48], and they modulate the Th1/Th2 balance by affecting T-helper (Th) cells [49]. Therefore, statins are promising treatments for psoriasis from an immunological perspective, as they influence cell differentiation and proliferation, inflammation, and cytokine production. Many studies have demonstrated improvements in psoriasis severity with statin use [8].

Most studies investigating the relationship between statins and psoriasis have used lipophilic statins (e.g., atorvastatin, simvastatin), which can readily enter hepatocytes and extrahepatic cells via passive diffusion. Limited studies have investigated hydrophilic statins (e.g., rosuvastatin, pravastatin), which require protein transporters for hepatocyte entry to inhibit HMG-CoA reductase [24]. Our study showed that hydrophilic statins with hepatoselectivity have a similar impact on psoriasis as lipophilic statins. This finding suggests that statins influence psoriasis through their immune-regulating functions via mevalonate pathway products or dendritic cell maturation, and their cholesterol-lowering functions in the liver. Psoriasis is closely associated with lipid abnormalities, as evidenced by a large meta-analysis showing significantly higher total cholesterol and LDL in patients with psoriasis compared to the control group [50]. An increase in oxidized modified lipoprotein (ox-LDL) in patients with psoriasis induces cell death through inflammation and cholesterol accumulation in lysosomes, promoting IL-23 expression through the inducible lectin-like ox-LDL receptor 1 (LOX-1) [45]. Lipid-lowering agents such as fish oil and fibrates have been shown to improve psoriasis [8], and recently, PCSK9 inhibitors (small interfering RNA that targets proprotein convertase subtilisin/kexin type 9) using gene silencing technologies have demonstrated efficacy in treating hyperlipidemia and psoriasis [25,51]. Therefore, cholesterol-lowering by statins may play a significant role in the development of psoriasis. However, the underlying immune regulation mechanisms of statins are not fully understood, and their anti-inflammatory effects must also be considered [10]. Future studies are needed to determine the relationship between lipid-lowering agents, particularly hydrophilic statins, and psoriasis.

Our study has several limitations. First, the cumulative days of statin use may not be entirely accurate, as the use of statins was estimated based on prescription information from health claim data. Second, individuals who take statins are more likely to be healthier than nonusers due to the healthy user effect, which could influence the incidence of psoriasis independently of the actual effects of statins. Third, the study population was limited to Koreans, and as such, there may be racial and regional differences in the relationship between statin use and the incidence of psoriasis. Fourth, although we adjusted for various variables to minimize confounding effects, unmeasured confounding factors may still exist due to the inherent limitations of retrospective studies. For example, vitamin D, which plays a crucial role in regulating inflammation and immune system function, has been linked to an increased risk of developing obesity, dyslipidemia, and psoriasis when deficient [52,53]. Furthermore, we did not investigate the concomitant use of other lipid-lowering agents, such as fish oil and fibrates, and we could not obtain information on their concurrent use. Last, the National Health Insurance data do not include PASI scores, limiting our ability to assess the severity and progression of psoriasis. Similarly, the absence of genetic data prevented us from investigating genetic backgrounds such as family history or HLA-C*06 allele, which could differentiate between early-onset and late-onset psoriasis [54].

Despite these limitations, our study has several strengths. One of these is analysing a large cohort database that includes demographic, socioeconomic, past medical, and lifestyle factors with a long-term follow-up period of 18 years. We adjusted for various confounders to minimize potential bias. Additionally, we applied propensity score overlap weights and conducted varied subgroup analyses to reduce the risk of selection bias and enhance the generalizability of our results.

## 5. Conclusions

In conclusion, our results revealed a significant association between statin use and a lower incidence of psoriasis during any period. Our findings also demonstrated that this association was significant for both lipophilic and hydrophilic statins. Future studies should investigate the underlying mechanisms of hydrophilic statins in psoriasis prevention and explore their potential applications in broader populations to address the limitations of this study.

## Figures and Tables

**Table 1:** General characteristics of participants before and after overlap propensity score weighting.

Characteristics		Before Overlap Weighting Adjustment			After Overlap Weighting Adjustment		
		Psoriasis	Control	Standardized Difference	Psoriasis	Control	Standardized Difference
Age (*n*, %)				0.00			0.00
	40–44	106 (1.28)	424 (1.28)		84 (1.28)	84 (1.28)	
	45–49	587 (7.09)	2348 (7.09)		466 (7.08)	466 (7.08)	
	50–54	1203 (14.52)	4812 (14.52)		953 (14.49)	953 (14.49)	
	55–59	1504 (18.15)	6016 (18.15)		1196 (18.18)	1196 (18.18)	
	60–64	1457 (17.59)	5828 (17.59)		1157 (17.58)	1157 (17.58)	
	65–69	1270 (15.33)	5080 (15.33)		1007 (15.30)	1007 (15.30)	
	70–74	1072 (12.94)	4288 (12.94)		852 (12.95)	852 (12.95)	
	75–79	678 (8.18)	2712 (8.18)		539 (8.19)	539 (8.19)	
	80–84	302 (3.65)	1208 (3.65)		241 (3.67)	242 (3.67)	
	85+	106 (1.28)	424 (1.28)		85 (1.29)	85 (1.29)	
Sex (*n*, %)				0.00			0.00
	Male	4649 (56.11)	18,596 (56.11)		3690 (56.07)	3690 (56.08)	
	Female	3636 (43.89)	14,544 (43.89)		2890 (43.93)	2890 (43.92)	
Income (*n*, %)				0.00			0.00
	1 (lowest)	1397 (16.86)	5588 (16.86)		1108 (16.84)	1108 (16.84)	
	2	1098 (13.25)	4392 (13.25)		872 (13.25)	872 (13.25)	
	3	1314 (15.86)	5256 (15.86)		1045 (15.88)	1045 (15.88)	
	4	1705 (20.58)	6820 (20.58)		1354 (20.58)	1354 (20.58)	
	5 (highest)	2771 (33.45)	11,084 (33.45)		2201 (33.45)	2201 (33.45)	
Region of residence (*n*, %)				0.00			0.00
	Urban	3445 (41.58)	13,780 (41.58)		2735 (41.57)	2736 (41.57)	
	Rural	4840 (58.42)	19,360 (58.42)		3845 (58.43)	3845 (58.43)	
Obesity † (*n*, %)				0.11			0.00
	Underweight	133 (1.61)	681 (2.05)		111 (1.68)	111 (1.68)	
	Normal	2478 (29.91)	11,118 (33.55)		2016 (30.63)	2016 (30.63)	
	Overweight	2201 (26.57)	9302 (28.07)		1771 (26.91)	1771 (26.91)	
	Obese I	3166 (38.21)	11,013 (33.23)		2447 (37.19)	2448 (37.19)	
	Obese II	307 (3.71)	1026 (3.10)		235 (3.58)	235 (3.58)	
Smoking status (*n*, %)				0.06			0.00
	Nonsmoker	5230 (63.13)	21,927 (66.16)		4197 (63.78)	4197 (63.78)	
	Past smoker	1378 (16.63)	5302 (16.00)		1086 (16.50)	1086 (16.50)	
	Current smoker	1677 (20.24)	5911 (17.84)		1298 (19.72)	1298 (19.72)	
Alcohol consumption (*n*, %)				0.01			0.00
	<1 time a week	5699 (68.79)	22,613 (68.23)		4521 (68.70)	4521 (68.70)	
	≥1 time a week	2586 (31.21)	10,527 (31.77)		2060 (31.30)	2060 (31.30)	0.00
SBP (Mean, SD)		127.73 (16.17)	127.39 (16.42)	0.02	127.68 (14.41)	127.68 (7.33)	0.00
DBP (Mean, SD)		78.51 (10.50)	78.43 (10.49)	0.01	78.50 (9.35)	78.50 (4.68)	0.00
Fasting blood glucose (Mean, SD)		103.08 (27.69)	102.54 (30.54)	0.02	102.96 (24.70)	102.96 (13.06)	0.00
CCI score (Mean, SD)		0.91 (1.54)	0.85 (1.53)	0.04	0.90 (1.35)	0.90 (0.71)	0.00
Diabetes history (*n*, %)		3569 (43.08)	11,814 (35.65)	0.15	2732 (41.52)	2732 (41.52)	0.00
Any statin (*n*, %)				0.16			0.13
	Nonuser	5623 (67.87)	24,888 (75.10)		4484 (68.14)	4864 (73.91)	
	Short-term user	1289 (15.56)	3818 (11.52)		1018 (15.47)	783 (11.90)	
	Long-term user	1373 (16.57)	4434 (13.38)		1079 (16.39)	934 (14.19)	
Lipophilic statin (*n*, %)				0.14			0.13
	Nonuser	6043 (72.94)	26,240 (79.18)		4814 (73.16)	5143 (78.15)	
	Short-term user	1224 (14.77)	3543 (10.69)		966 (14.69)	732 (11.12)	
	Long-term user	1018 (12.29)	3357 (10.13)		799 (12.15)	706 (10.73)	
Hydrophilic statin (*n*, %)				0.07			0.05
	Nonuser	7578 (91.47)	30,937 (93.35)		6023 (91.53)	6122 (93.03)	
	Short-term user	409 (4.94)	1253 (3.78)		323 (4.90)	258 (3.92)	
	Long-term user	298 (3.60)	950 (2.87)		235 (3.56)	201 (3.06)	

Abbreviations: CCI, Charlson Comorbidity Index; SBP, Systolic blood pressure; DBP, Diastolic blood pressure; † Obesity (BMI, body mass index, kg/m^2^) was categorized as <18.5 (underweight), ≥18.5 to <23 (normal), ≥23 to <25 (overweight), ≥25 to <30 (obese I), and ≥30 (obese II).

**Table 2:** Crude and adjusted odds ratios (95% confidence intervals) of any statin/lipophilic statin/hydrophilic statin for psoriasis.

Characteristics		N of Psoriasis	N of Control	Odd Ratios for Psoriasis (95% Confidence Interval)			
		(Exposure/Total, %)	(Exposure/Total, %)	Crude	*p* Value	Overlap Weighted Model †	*p* Value
Any statin prescription							
	Nonuser	5623/8285 (67.87)	24,888/33,140 (75.1)	1		1	
	Short-term user	1289/8285 (15.56)	3818/33,140 (11.52)	1.49 (1.39–1.60)	<0.001	0.70 (0.66–0.74)	<0.001
	Long-term user	1373/8285 (16.57)	4434/33,140 (13.38)	1.37 (1.28–1.47)	<0.001	0.77 (0.73–0.82)	<0.001
Lipophilic statin prescription							
	Nonuser	6043/8285 (72.94)	26,240/33,140 (79.18)	1		1	
	Short-term user	1224/8285 (14.77)	3543/33,140 (10.69)	1.50 (1.40–1.61)	<0.001	0.70 (0.66–0.74)	<0.001
	Long-term user	1018/8285 (12.29)	3357/33,140 (10.13)	1.32 (1.22–1.42)	<0.001	0.81 (0.76–0.86)	<0.001
Hydrophilic statin prescription							
	Nonuser	7578/8285 (91.47)	30,937/33,140 (93.35)	1	A	1	
	Short-term user	409/8285 (4.93)	1253/33,140 (3.78)	1.33 (1.19–1.49)	<0.001	0.78 (0.71–0.86)	<0.001
	Long-term user	298/8285 (3.6)	950/33,140 (2.87)	1.28 (1.12–1.46)	<0.001	0.84 (0.75–0.94)	0.002

† Adjusted for age, sex, income, region of residence, SBP, DBP, fasting blood glucose, obesity, smoking, alcohol consumption, diabetes history, and CCI scores.

## Data Availability

Restrictions apply to the data availability. Data were obtained from the Korean National Health Insurance Sharing Service (NHISS) and are available at https://nhiss.nhis.or.kr (accessed on 25 January 2022) with permission from the NHISS.

## Supplementary Materials

The following supporting information can be downloaded at: https://www.mdpi.com/article/10.3390/jcm14010091/s1, Table S1: Crude and overlap propensity score weighted odd ratios of dates of any statin prescription for psoriasis; Table S2: Crude and overlap propensity score weighted odd ratios of dates of lipophilic statin prescription for psoriasis; Table S3: Crude and overlap propensity score weighted odd ratios of dates of hydrophilic statin prescription for psoriasis.

## References

[1] Michalek I., Loring B., John S. (2016). WHO Global Report on Psoriasis.

[2] Lee J.Y., Kang S., Park J.S., Jo S.J. (2017). Prevalence of Psoriasis in Korea: A Population-Based Epidemiological Study Using the Korean National Health Insurance Database. Ann. Dermatol..

[3] Raychaudhuri S.P., Farber E.M. (2001). The prevalence of psoriasis in the world. J. Eur. Acad. Dermatol. Venereol..

[4] Takeshita J., Grewal S., Langan S.M., Mehta N.N., Ogdie A., Van Voorhees A.S., Gelfand J.M. (2017). Psoriasis and comorbid diseases: Epidemiology. J. Am. Acad. Dermatol..

[5] Dhabale A., Nagpure S. (2022). Types of Psoriasis and Their Effects on the Immune System. Cureus.

[6] Javitz H.S., Ward M.M., Farber E., Nail L., Vallow S.G. (2002). The direct cost of care for psoriasis and psoriatic arthritis in the United States. J. Am. Acad. Dermatol..

[7] Elmets C.A., Leonardi C.L., Davis D.M.R., Gelfand J.M., Lichten J., Mehta N.N., Armstrong A.W., Connor C., Cordoro K.M., Elewski B.E. (2019). Joint AAD-NPF guidelines of care for the management and treatment of psoriasis with awareness and attention to comorbidities. J. Am. Acad. Dermatol..

[8] Wang J., Zhang S., Xing M., Hong S., Liu L., Ding X.J., Sun X.Y., Luo Y., Wang C.X., Zhang M. (2022). Current evidence on the role of lipid lowering drugs in the treatment of psoriasis. Front. Med..

[9] Vagelos P.R. (1991). Are prescription drug prices high?. Science.

[10] Diamantis E., Kyriakos G., Quiles-Sanchez L.V., Farmaki P., Troupis T. (2017). The Anti-Inflammatory Effects of Statins on Coronary Artery Disease: An Updated Review of the Literature. Curr. Cardiol. Rev..

[11] Dehnavi S., Sohrabi N., Sadeghi M., Lansberg P., Banach M., Al-Rasadi K., Johnston T.P., Sahebkar A. (2020). Statins and autoimmunity: State-of-the-art. Pharmacol. Ther..

[12] Willey J.Z., Elkind M.S. (2010). 3-Hydroxy-3-methylglutaryl-coenzyme A reductase inhibitors in the treatment of central nervous system diseases. Arch. Neurol..

[13] Collins R., Armitage J., Parish S., Sleight P., Peto R. (2004). Effects of cholesterol-lowering with simvastatin on stroke and other major vascular events in 20,536 people with cerebrovascular disease or other high-risk conditions. Lancet.

[14] Wahner A.D., Bronstein J.M., Bordelon Y.M., Ritz B. (2008). Statin use and the risk of Parkinson disease. Neurology.

[15] Youssef S., Stüve O., Patarroyo J.C., Ruiz P.J., Radosevich J.L., Hur E.M., Bravo M., Mitchell D.J., Sobel R.A., Steinman L. (2002). The HMG-CoA reductase inhibitor, atorvastatin, promotes a Th2 bias and reverses paralysis in central nervous system autoimmune disease. Nature.

[16] Vernaglione L., Cristofano C., Muscogiuri P., Chimienti S. (2004). Does atorvastatin influence serum C-reactive protein levels in patients on long-term hemodialysis?. Am. J. Kidney Dis..

[17] Sindhu S., Singh H.K., Salman M.T., Fatima J., Verma V.K. (2011). Effects of atorvastatin and rosuvastatin on high-sensitivity C-reactive protein and lipid profile in obese type 2 diabetes mellitus patients. J. Pharmacol. Pharmacother..

[18] Macin S.M., Perna E.R., Farías E.F., Franciosi V., Cialzeta J.R., Brizuela M., Medina F., Tajer C., Doval H., Badaracco R. (2005). Atorvastatin has an important acute anti-inflammatory effect in patients with acute coronary syndrome: Results of a randomized, double-blind, placebo-controlled study. Am. Heart J..

[19] Naseri M., Hadipour A., Sepaskhah M., Namazi M.R. (2010). The remarkable beneficial effect of adding oral simvastatin to topical betamethasone for treatment of psoriasis: A double-blind, randomized, placebo-controlled study. Niger. J. Med..

[20] Ramessur R., Gill D. (2017). The effect of statins on severity of psoriasis: A systematic review. Indian. J. Dermatol. Venereol. Leprol..

[21] Yamamoto M., Ikeda M., Kodama H., Sano S. (2008). Transition of psoriasiform drug eruption to psoriasis de novo evidenced by histopathology. J. Dermatol..

[22] Jacobi T.C., Highet A. (2003). A clinical dilemma while treating hypercholesterolaemia in psoriasis. Br. J. Dermatol..

[23] Chodick G., Weitzman D., Shalev V., Weil C., Amital H. (2015). Adherence to statins and the risk of psoriasis: A population-based cohort study. Br. J. Dermatol..

[24] Climent E., Benaiges D., Pedro-Botet J. (2021). Hydrophilic or Lipophilic Statins?. Front. Cardiovasc. Med..

[25] Zhao S.S., Yiu Z.Z.N., Barton A., Bowes J. (2023). Association of Lipid-Lowering Drugs With Risk of Psoriasis: A Mendelian Randomization Study. JAMA Dermatol..

[26] Shenoy C., Shenoy M.M., Rao G.K. (2015). Dyslipidemia in Dermatological Disorders. N. Am. J. Med. Sci..

[27] Seong S.C., Kim Y.Y., Park S.K., Khang Y.H., Kim H.C., Park J.H., Kang H.J., Do C.H., Song J.S., Lee E.J. (2017). Cohort profile: The National Health Insurance Service-National Health Screening Cohort (NHIS-HEALS) in Korea. BMJ Open.

[28] Mantel-Teeuwisse A.K., Goettsch W.G., Klungel O.H., de Boer A., Herings R.M. (2004). Long term persistence with statin treatment in daily medical practice. Heart.

[29] Kim J.H., Chang I.B., Kim Y.H., Kwon M.J., Kim J.H., Choi H.G. (2022). Association between statin use and Parkinson’s disease in Korean patients with hyperlipidemia. Park. Relat. Disord..

[30] Ham S.P., Oh J.H., Park H.J., Kim J.U., Kim H.Y., Jung S.Y., Choi S.Y., Seol J.E., Kim H., Kim M.S. (2020). Validity of Diagnostic Codes for Identification of Psoriasis Patients in Korea. Ann. Dermatol..

[31] Quan H., Li B., Couris C.M., Fushimi K., Graham P., Hider P., Januel J.M., Sundararajan V. (2011). Updating and validating the Charlson comorbidity index and score for risk adjustment in hospital discharge abstracts using data from 6 countries. Am. J. Epidemiol..

[32] Thomas L.E., Li F., Pencina M.J. (2020). Overlap Weighting: A Propensity Score Method That Mimics Attributes of a Randomized Clinical Trial. JAMA.

[33] Aleid A.M., Almutairi G., Alrizqi R., Nukaly H.Y., Alkhanani J.J., AlHuraish D.S., Alshanti H.Y., Algaidi Y.S., Alyami H., Alrasheeday A. (2024). The Impact of Statins on Disease Severity and Quality of Life in Patients with Psoriasis: A Systematic Review and Meta-Analysis. Healthcare.

[34] Brauchli Y.B., Jick S.S., Meier C.R. (2011). Statin use and risk of first-time psoriasis diagnosis. J. Am. Acad. Dermatol..

[35] Langan S.M., Seminara N.M., Shin D.B., Troxel A.B., Kimmel S.E., Mehta N.N., Margolis D.J., Gelfand J.M. (2012). Prevalence of metabolic syndrome in patients with psoriasis: A population-based study in the United Kingdom. J. Investig. Dermatol..

[36] de Alcantara C.C., Reiche E.M.V., Simão A.N.C. (2021). Cytokines in psoriasis. Adv. Clin. Chem..

[37] Cibrian D., de la Fuente H., Sánchez-Madrid F. (2020). Metabolic Pathways That Control Skin Homeostasis and Inflammation. Trends Mol. Med..

[38] Davidovici B.B., Sattar N., Prinz J., Puig L., Emery P., Barker J.N., van de Kerkhof P., Ståhle M., Nestle F.O., Girolomoni G. (2010). Psoriasis and systemic inflammatory diseases: Potential mechanistic links between skin disease and co-morbid conditions. J. Investig. Dermatol..

[39] Alajroush W.A., Alrshid A.I., Alajlan A.H., Alsalamah Y.B., Alhumaidan M.I., Alhoumedan A.I., Alrasheed M.I., Alowairdhi Y.A., Alowirdi F., Aljoufi A.Z. (2024). Psoriasis and Metabolic Disorders: A Comprehensive Meta-Analysis of Million Adults Worldwide. Cureus.

[40] Mirghani H., Altemani A.T., Altemani S.T., Alhatlani J.A.A., Alsulaimani N.M.I., AlHuraish D.S.A., Al Mudhi A.H.A., Ghabban W.J.R., Alanazi A.H., Alamrani B.A. (2023). The Cross Talk Between Psoriasis, Obesity, and Dyslipidemia: A Meta-Analysis. Cureus.

[41] Wang J., Yu Y., Liu L., Wang C., Sun X., Zhou Y., Hong S., Cai X., Xu W., Li X. (2024). Global prevalence of obesity in patients with psoriasis: An analysis in the past two decades. Autoimmun. Rev..

[42] Huerta C., Rivero E., Rodríguez L.A. (2007). Incidence and risk factors for psoriasis in the general population. Arch. Dermatol..

[43] Ma L., Li M., Wang H., Li Y., Bai B. (2014). High prevalence of cardiovascular risk factors in patients with moderate or severe psoriasis in northern China. Arch. Dermatol. Res..

[44] Salihbegovic E.M., Kurtalic N., Omerkic E. (2021). Smoking Cigarettes and Consuming Alcohol in Patients with Psoriasis. Mater. Sociomed..

[45] Nowowiejska J., Baran A., Flisiak I. (2021). Aberrations in Lipid Expression and Metabolism in Psoriasis. Int. J. Mol. Sci..

[46] Pietrzak A., Chabros P., Grywalska E., Kiciński P., Pietrzak-Franciszkiewicz K., Krasowska D., Kandzierski G. (2019). Serum lipid metabolism in psoriasis and psoriatic arthritis—An update. Arch. Med. Sci..

[47] Armstrong A.W., Read C. (2020). Pathophysiology, Clinical Presentation, and Treatment of Psoriasis: A Review. JAMA.

[48] Li H., Wang C.C., Zhang M., Li X.L., Zhang P., Yue L.T., Miao S., Wang S., Liu Y., Li Y.B. (2015). Statin-modified dendritic cells regulate humoral immunity in experimental autoimmune myasthenia gravis. Mol. Cell. Neurosci..

[49] Ntolkeras G., Barba C., Mavropoulos A., Vasileiadis G.K., Dardiotis E., Sakkas L.I., Hadjigeorgiou G., Bogdanos D.P. (2019). On the immunoregulatory role of statins in multiple sclerosis: The effects on Th17 cells. Immunol. Res..

[50] Ramezani M., Zavattaro E., Sadeghi M. (2019). Evaluation of serum lipid, lipoprotein, and apolipoprotein levels in psoriatic patients: A systematic review and meta-analysis of case-control studies. Postep. Dermatol. Alergol..

[51] Di Fusco S.A., Maggioni A.P., Bernelli C., Perone F., De Marzo V., Conte E., Musella F., Uccello G., Luca L., Gabrielli D. (2022). Inclisiran: A New Pharmacological Approach for Hypercholesterolemia. Rev. Cardiovasc. Med..

[52] Bakar R.S., Jaapar S.Z.S., Azmi A.F., Aun Y.C. (2021). Depression and anxiety among patients with psoriasis: A correlation with quality of life and associated factors. J. Taibah Univ. Med. Sci..

[53] Karampinis E., Goudouras G., Ntavari N., Bogdanos D.P., Roussaki-Schulze A.V., Zafiriou E. (2023). Serum vitamin D levels can be predictive of psoriasis flares up after COVID-19 vaccination: A retrospective case control study. Front. Med..

[54] Queiro R., Tejón P., Alonso S., Coto P. (2014). Age at disease onset: A key factor for understanding psoriatic disease. Rheumatology.

