# Supplementary material for: Association Between Statin Use and Psoriasis in Patients with Dyslipidemia: A Korean National Health Screening Cohort Study
Source: J Clin Med. 2024 Dec 27;14(1):91. doi: 10.3390/jcm14010091 (PMC11721600; doi:10.3390/jcm14010091)
Supplement: Supplementary file 1 [file jcm-14-00091-s001.zip › jcm-3271089-supplementary.pdf]

Journal of Clinical Medicine

Table S1. Crude and overlap propensity score weighted odd ratios of dates of any statin prescription for psoriasis.

Characteristics	N of Psoriasis	N of Control	Odd Ratios for Psoriasis (95% Confidence Interval)			
	(Exposure/Total, %)	(Exposure/Total, %)	Crude	*p*-Value	Overlap Weighted Model †	*p*-Value
Age < 65 years old (n= 24,285)						
Nonuser	3,660/4,857 (75.36)	15,692/19,428 (80.77)	1		1	
Short-term user	657/4,857 (13.53)	2,061/19,428 (10.61)	1.37 (1.24-1.50)	<0.001*	0.78 (0.72-0.84)	<0.001*
Long-term user	540/4,857 (11.12)	1,675/19,428 (8.62)	1.38 (1.25-1.53)	<0.001*	0.79 (0.72-0.86)	<0.001*
Age ≥ 65 years old (n= 17,140)						
Nonuser	1,963/3,428 (57.26)	9,196/13,712 (67.07)	1		1	
Short-term user	632/3,428 (18.44)	1,757/13,712 (12.81)	1.69 (1.52-1.87)	<0.001*	0.62 (0.57-0.67)	<0.001*
Long-term user	833/3,428 (24.3)	2,759/13,712 (20.12)	1.41 (1.29-1.55)	<0.001*	0.76 (0.70-0.82)	<0.001*
Male (n= 23,245)						
Nonuser	3,239/4,649 (69.67)	14,363/18,596 (77.24)	1		1	
Short-term user	641/4,649 (13.79)	1,886/18,596 (10.14)	1.51 (1.37-1.66)	<0.001*	0.70 (0.64-0.76)	<0.001*
Long-term user	769/4,649 (16.54)	2,347/18,596 (12.62)	1.45 (1.33-1.59)	<0.001*	0.74 (0.68-0.80)	<0.001*
Female (n= 18,180)						
Nonuser	2,384/3,636 (65.57)	10,525/14,544 (72.37)	1		1	
Short-term user	648/3,636 (17.82)	1,932/14,544 (13.28)	1.48 (1.34-1.64)	<0.001*	0.70 (0.64-0.76)	<0.001*
Long-term user	604/3,636 (16.61)	2,087/14,544 (14.35)	1.28 (1.16-1.41)	<0.001*	0.81 (0.75-0.89)	<0.001*
Low income groups (n= 19,045)						
Nonuser	2,587/3,809 (67.92)	11,605/15,236 (76.17)	1		1	
Short-term user	601/3,809 (15.78)	1,734/15,236 (11.38)	1.56 (1.40-1.72)	<0.001*	0.67 (0.62-0.73)	<0.001*
Long-term user	621/3,809 (16.3)	1,897/15,236 (12.45)	1.47 (1.33-1.62)	<0.001*	0.72 (0.66-0.78)	<0.001*
High income groups (n= 22,380)						
Nonuser	3,036/4,476 (67.83)	13,283/17,904 (74.19)	1		1	
Short-term user	688/4,476 (15.37)	2,084/17,904 (11.64)	1.44 (1.31-1.59)	<0.001*	0.72 (0.67-0.78)	<0.001*
Long-term user	752/4,476 (16.8)	2,537/17,904 (14.17)	1.30 (1.18-1.42)	<0.001*	0.82 (0.76-0.89)	<0.001*
Urban residents (n= 17,225)						
Nonuser	2,297/3,445 (66.68)	10,227/13,780 (74.22)	1		1	
Short-term user	535/3,445 (15.53)	1,601/13,780 (11.62)	1.49 (1.34-1.66)	<0.001*	0.70 (0.64-0.76)	<0.001*
Long-term user	613/3,445 (17.79)	1,952/13,780 (14.17)	1.40 (1.26-1.55)	<0.001*	0.75 (0.69-0.82)	<0.001*
Rural residents (n= 24,200)						
Nonuser	3,326/4,840 (68.72)	14,661/19,360 (75.73)	1		1	
Short-term user	754/4,840 (15.58)	2,217/19,360 (11.45)	1.50 (1.37-1.64)	<0.001*	0.70 (0.65-0.75)	<0.001*
Long-term user	760/4,840 (15.7)	2,482/19,360 (12.82)	1.35 (1.23-1.48)	<0.001*	0.79 (0.73-0.85)	<0.001*
Underweight (n= 814)						
Nonuser	102/133 (76.69)	593/681 (87.08)	1		1	
Short-term user	14/133 (10.53)	48/681 (7.05)	1.70 (0.90-3.19)	0.101	0.58 (0.35-0.96)	0.035*
Long-term user	17/133 (12.78)	40/681 (5.87)	2.47 (1.35-4.53)	0.003*	0.37 (0.21-0.63)	<0.001*
Normal weight (n= 13,596)						
Nonuser	1,769/2,478 (71.39)	8,978/11,118 (80.75)	1		1	
Short-term user	392/2,478 (15.82)	1,075/11,118 (9.67)	1.85 (1.63-2.10)	<0.001*	0.56 (0.50-0.62)	<0.001*
Long-term user	317/2,478 (12.79)	1,065/11,118 (9.58)	1.51 (1.32-1.73)	<0.001*	0.70 (0.63-0.78)	<0.001*
Overweight (n= 11,503)						
Nonuser	1,528/2,201 (69.42)	6,962/9,302 (74.84)	1		1	
Short-term user	306/2,201 (13.9)	1,097/9,302 (11.79)	1.27 (1.11-1.46)	<0.001*	0.81 (0.72-0.90)	<0.001*

Long-term user	367/2,201 (16.67)	1,243/9,302 (13.36)	1.35 (1.18-1.53)	<0.001*	0.76 (0.68-0.85)	<0.001*
Obese (n= 15,512)						
Nonuser	2,224/3,473 (64.04)	8,355/12,039 (69.4)	1		1	
Short-term user	577/3,473 (16.61)	1,598/12,039 (13.27)	1.36 (1.22-1.51)	<0.001*	0.75 (0.68-0.82)	<0.001*
Long-term user	672/3,473 (19.35)	2,086/12,039 (17.33)	1.21 (1.10-1.34)	<0.001*	0.83 (0.77-0.91)	<0.001*
Non-smoker (n= 27,157)						
Nonuser	3,506/5,230 (67.04)	16,324/21,927 (74.45)	1		1	
Short-term user	861/5,230 (16.46)	2,663/21,927 (12.14)	1.51 (1.38-1.64)	<0.001*	0.70 (0.65-0.75)	<0.001*
Long-term user	863/5,230 (16.5)	2,940/21,927 (13.41)	1.37 (1.26-1.49)	<0.001*	0.79 (0.74-0.85)	<0.001*
Past and current smoker (n= 14,268)						
Nonuser	2,117/3,055 (69.3)	8,564/11,213 (76.38)	1		1	
Short-term user	428/3,055 (14.01)	1,155/11,213 (10.3)	1.50 (1.33-1.69)	<0.001*	0.69 (0.62-0.77)	<0.001*
Long-term user	510/3,055 (16.69)	1,494/11,213 (13.32)	1.38 (1.24-1.54)	<0.001*	0.74 (0.67-0.82)	<0.001*
Alcohol consumption <1 time a week (n= 28,312)						
Nonuser	3,788/5,699 (66.47)	16,695/22,613 (73.83)	1		1	
Short-term user	924/5,699 (16.21)	2,742/22,613 (12.13)	1.49 (1.37-1.61)	<0.001*	0.70 (0.65-0.75)	<0.001*
Long-term user	987/5,699 (17.32)	3,176/22,613 (14.05)	1.37 (1.26-1.48)	<0.001*	0.77 (0.72-0.82)	<0.001*
Alcohol consumption ≥1 time a week (n= 13,113)						
Nonuser	1,835/2,586 (70.96)	8,193/10,527 (77.83)	1		1	
Short-term user	365/2,586 (14.11)	1,076/10,527 (10.22)	1.51 (1.33-1.72)	<0.001*	0.70 (0.63-0.78)	<0.001*
Long-term user	386/2,586 (14.93)	1,258/10,527 (11.95)	1.37 (1.21-1.55)	<0.001*	0.79 (0.71-0.88)	<0.001*
SBP < 120 mmHg and DBP < 80 mmHg (n= 10,587)						
Nonuser	1,436/2,040 (70.39)	6,706/8,547 (78.46)	1		1	
Short-term user	288/2,040 (14.12)	865/8,547 (10.12)	1.56 (1.35-1.80)	<0.001*	0.66 (0.59-0.75)	<0.001*
Long-term user	316/2,040 (15.49)	976/8,547 (11.42)	1.51 (1.32-1.74)	<0.001*	0.70 (0.62-0.79)	<0.001*
SBP ≥ 120 mmHg or DBP ≥ 80 mmHg (n= 30,838)						
Nonuser	4,187/6,245 (67.05)	18,182/24,593 (73.93)	1		1	
Short-term user	1,001/6,245 (16.03)	2,953/24,593 (12.01)	1.47 (1.36-1.59)	<0.001*	0.71 (0.66-0.76)	<0.001*
Long-term user	1,057/6,245 (16.93)	3,458/24,593 (14.06)	1.33 (1.23-1.43)	<0.001*	0.80 (0.75-0.85)	<0.001*
Fasting blood glucose < 100 mg/dL (n= 24,301)						
Nonuser	3,413/4,725 (72.23)	15,587/19,576 (79.62)	1		1	
Short-term user	691/4,725 (14.62)	2,037/19,576 (10.41)	1.55 (1.41-1.70)	<0.001*	0.67 (0.62-0.73)	<0.001*
Long-term user	621/4,725 (13.14)	1,952/19,576 (9.97)	1.45 (1.32-1.60)	<0.001*	0.73 (0.67-0.79)	<0.001*
Fasting blood glucose ≥ 100 mg/dL (n= 17,124)						
Nonuser	2,210/3,560 (62.08)	9,301/13,564 (68.57)	1		1	
Short-term user	598/3,560 (16.8)	1,781/13,564 (13.13)	1.41 (1.27-1.57)	<0.001*	0.73 (0.67-0.80)	<0.001*
Long-term user	752/3,560 (21.12)	2,482/13,564 (18.3)	1.28 (1.16-1.40)	<0.001*	0.82 (0.76-0.89)	<0.001*
CCI scores = 0 (n= 26,021)						
Nonuser	3,540/4,941 (71.65)	16,351/21,080 (77.57)	1		1	
Short-term user	706/4,941 (14.29)	2,248/21,080 (10.66)	1.45 (1.32-1.59)	<0.001*	0.72 (0.67-0.78)	<0.001*
Long-term user	695/4,941 (14.07)	2,481/21,080 (11.77)	1.29 (1.18-1.42)	<0.001*	0.83 (0.77-0.89)	<0.001*
CCI scores = 1 (n= 6,679)						
Nonuser	916/1,483 (61.77)	3,637/5,196 (70)	1		1	
Short-term user	257/1,483 (17.33)	687/5,196 (13.22)	1.49 (1.27-1.74)	<0.001*	0.68 (0.60-0.79)	<0.001*
Long-term user	310/1,483 (20.9)	872/5,196 (16.78)	1.41 (1.22-1.64)	<0.001*	0.71 (0.62-0.81)	<0.001*
CCI scores ≥ 2 (n= 8,725)						
Nonuser	1,167/1,861 (62.71)	4,900/6,864 (71.39)	1		1	
Short-term user	326/1,861 (17.52)	883/6,864 (12.86)	1.55 (1.34-1.79)	<0.001*	0.67 (0.59-0.75)	<0.001*
Long-term user	368/1,861 (19.77)	1,081/6,864 (15.75)	1.43 (1.25-1.64)	<0.001*	0.73 (0.65-0.81)	<0.001*
Non diabetes history (n= 26,042)						
Nonuser	3,547/4,716 (75.21)	17,387/21,326 (81.53)	1		1	
Short-term user	619/4,716 (13.13)	2,036/21,326 (9.55)	1.49 (1.35-1.64)	<0.001*	0.68 (0.63-0.73)	<0.001*
Long-term user	550/4,716 (11.66)	1,903/21,326 (8.92)	1.42 (1.28-1.57)	<0.001*	0.71 (0.66-0.77)	<0.001*

Diabetes history (n= 15,383)						
Nonuser	2,076/3,569 (58.17)	7,501/11,814 (63.49)	1		1	
Short-term user	670/3,569 (18.77)	1,782/11,814 (15.08)	1.36 (1.23-1.50)	<0.001*	0.72 (0.66-0.79)	<0.001*
Long-term user	823/3,569 (23.06)	2,531/11,814 (21.42)	1.17 (1.07-1.29)	<0.001*	0.83 (0.76-0.89)	<0.001*

Abbreviations: CCI, Charlson Comorbidity Index; SBP, Systolic blood pressure; DBP, Diastolic blood pressure; * Significance at P < 0.05. † Adjusted for age, sex, income, region of residence, SBP, DBP, fasting blood glucose, obesity, smoking, alcohol consumption, diabetes history, and CCI scores.

Table S2. Crude and overlap propensity score weighted odd ratios of dates of lipophilic statin prescription for psoriasis.

**Characteristics**	**N of Psoriasis**	**N of Control**	**Odd ratios for Psoriasis (95% Confidence Interval)**			
	**(Exposure/Total, %)**	**(Exposure/Total, %)**	**Crude**	***p*-Value**	**Overlap Weighted Model †**	***p*-Value**
Age < 65 years old (n= 24,285)						
Nonuser	3,842/4,857 (79.1)	16,271/19,428 (83.75)	1		1	
Short-term user	604/4,857 (12.44)	1,871/19,428 (9.63)	1.37 (1.24-1.51)	<0.001*	0.78 (0.72-0.85)	<0.001*
Long-term user	411/4,857 (8.46)	1,286/19,428 (6.62)	1.35 (1.20-1.52)	<0.001*	0.80 (0.73-0.89)	<0.001*
Age ≥ 65 years old (n= 17,140)						
Nonuser	2,201/3,428 (64.21)	9,969/13,712 (72.7)	1		1	
Short-term user	620/3,428 (18.09)	1,672/13,712 (12.19)	1.68 (1.52-1.86)	<0.001*	0.62 (0.57-0.68)	<0.001*
Long-term user	607/3,428 (17.71)	2,071/13,712 (15.1)	1.33 (1.20-1.47)	<0.001*	0.81 (0.74-0.88)	<0.001*
Male (n= 23,245)						
Nonuser	3,488/4,649 (75.03)	15,119/18,596 (81.3)	1		1	
Short-term user	606/4,649 (13.04)	1,733/18,596 (9.32)	1.52 (1.37-1.67)	<0.001*	0.70 (0.64-0.76)	<0.001*
Long-term user	555/4,649 (11.94)	1,744/18,596 (9.38)	1.38 (1.25-1.53)	<0.001*	0.78 (0.72-0.85)	<0.001*
Female (n= 18,180)						
Nonuser	2,555/3,636 (70.27)	11,121/14,544 (76.46)	1		1	
Short-term user	618/3,636 (17)	1,810/14,544 (12.44)	1.49 (1.34-1.64)	<0.001*	0.70 (0.64-0.76)	<0.001*
Long-term user	463/3,636 (12.73)	1,613/14,544 (11.09)	1.25 (1.12-1.40)	<0.001*	0.83 (0.76-0.92)	<0.001*
Low income groups (n= 19,045)						
Nonuser	2,788/3,809 (73.2)	12,196/15,236 (80.05)	1		1	
Short-term user	569/3,809 (14.94)	1,599/15,236 (10.49)	1.56 (1.40-1.73)	<0.001*	0.67 (0.62-0.74)	<0.001*
Long-term user	452/3,809 (11.87)	1,441/15,236 (9.46)	1.37 (1.23-1.54)	<0.001*	0.78 (0.71-0.86)	<0.001*
High income groups (n= 22,380)						
Nonuser	3,255/4,476 (72.72)	14,044/17,904 (78.44)	1		1	
Short-term user	655/4,476 (14.63)	1,944/17,904 (10.86)	1.45 (1.32-1.60)	<0.001*	0.72 (0.66-0.78)	<0.001*
Long-term user	566/4,476 (12.65)	1,916/17,904 (10.7)	1.27 (1.15-1.41)	<0.001*	0.84 (0.77-0.91)	<0.001*
Urban residents (n= 17,225)						
Nonuser	2,476/3,445 (71.87)	10,834/13,780 (78.62)	1		1	
Short-term user	518/3,445 (15.04)	1,457/13,780 (10.57)	1.56 (1.39-1.74)	<0.001*	0.67 (0.61-0.74)	<0.001*
Long-term user	451/3,445 (13.09)	1,489/13,780 (10.81)	1.33 (1.18-1.49)	<0.001*	0.80 (0.72-0.88)	<0.001*
Rural residents (n= 24,200)						
Nonuser	3,567/4,840 (73.7)	15,406/19,360 (79.58)	1		1	
Short-term user	706/4,840 (14.59)	2,086/19,360 (10.77)	1.46 (1.33-1.60)	<0.001*	0.72 (0.67-0.78)	<0.001*
Long-term user	567/4,840 (11.71)	1,868/19,360 (9.65)	1.31 (1.19-1.45)	<0.001*	0.81 (0.75-0.89)	<0.001*
Underweight (n= 814)						
Nonuser	108/133 (81.2)	607/681 (89.13)	1		1	
Short-term user	13/133 (9.77)	44/681 (6.46)	1.66 (0.87-3.19)	0.127	0.58 (0.34-0.98)	0.044*
Long-term user	12/133 (9.02)	30/681 (4.41)	2.25 (1.12-4.53)	0.023*	0.40 (0.21-0.73)	0.003*
Normal weight (n= 13,596)						

Nonuser	1,874/2,478 (75.63)	9,324/11,118 (83.86)	1		1	
Short-term user	368/2,478 (14.85)	978/11,118 (8.8)	1.87 (1.64-2.13)	<0.001*	0.56 (0.50-0.62)	<0.001*
Long-term user	236/2,478 (9.52)	816/11,118 (7.34)	1.44 (1.23-1.68)	<0.001*	0.74 (0.65-0.84)	<0.001*
Overweight (n= 11,503)						
Nonuser	1,628/2,201 (73.97)	7,346/9,302 (78.97)	1		1	
Short-term user	304/2,201 (13.81)	1,007/9,302 (10.83)	1.36 (1.19-1.57)	<0.001*	0.76 (0.68-0.85)	<0.001*
Long-term user	269/2,201 (12.22)	949/9,302 (10.2)	1.28 (1.11-1.48)	<0.001*	0.81 (0.72-0.91)	<0.001*
Obese (n= 15,512)						
Nonuser	2,433/3,473 (70.05)	8,963/12,039 (74.45)	1		1	
Short-term user	539/3,473 (15.52)	1,514/12,039 (12.58)	1.31 (1.18-1.46)	<0.001*	0.78 (0.71-0.85)	<0.001*
Long-term user	501/3,473 (14.43)	1,562/12,039 (12.97)	1.18 (1.06-1.32)	0.003*	0.86 (0.78-0.95)	0.002*
Non-smoker (n= 27,157)						
Nonuser	3,764/5,230 (71.97)	17,194/21,927 (78.41)	1		1	
Short-term user	819/5,230 (15.66)	2,471/21,927 (11.27)	1.51 (1.39-1.65)	<0.001*	0.70 (0.65-0.75)	<0.001*
Long-term user	647/5,230 (12.37)	2,262/21,927 (10.32)	1.31 (1.19-1.44)	<0.001*	0.83 (0.77-0.90)	<0.001*
Past and current smoker (n= 14,268)						
Nonuser	2,279/3,055 (74.6)	9,046/11,213 (80.67)	1		1	
Short-term user	405/3,055 (13.26)	1,072/11,213 (9.56)	1.50 (1.33-1.70)	<0.001*	0.70 (0.63-0.77)	<0.001*
Long-term user	371/3,055 (12.14)	1,095/11,213 (9.77)	1.34 (1.19-1.53)	<0.001*	0.76 (0.69-0.85)	<0.001*
Alcohol consumption <1 time a week (n= 28,312)						
Nonuser	4,075/5,699 (71.5)	17,629/22,613 (77.96)	1		1	
Short-term user	897/5,699 (15.74)	2,554/22,613 (11.29)	1.52 (1.40-1.65)	<0.001*	0.69 (0.64-0.73)	<0.001*
Long-term user	727/5,699 (12.76)	2,430/22,613 (10.75)	1.29 (1.18-1.42)	<0.001*	0.81 (0.76-0.88)	<0.001*
Alcohol consumption ≥1 time a week (n= 13,113)						
Nonuser	1,968/2,586 (76.1)	8,611/10,527 (81.8)	1		1	
Short-term user	327/2,586 (12.65)	989/10,527 (9.39)	1.45 (1.27-1.65)	<0.001*	0.74 (0.66-0.83)	<0.001*
Long-term user	291/2,586 (11.25)	927/10,527 (8.81)	1.37 (1.19-1.58)	<0.001*	0.79 (0.71-0.89)	<0.001*
SBP < 120 mmHg and DBP < 80 mmHg (n= 10,587)						
Nonuser	1,550/2,040 (75.98)	7,032/8,547 (82.27)	1		1	
Short-term user	264/2,040 (12.94)	793/8,547 (9.28)	1.51 (1.30-1.75)	<0.001*	0.69 (0.61-0.78)	<0.001*
Long-term user	226/2,040 (11.08)	722/8,547 (8.45)	1.42 (1.21-1.67)	<0.001*	0.76 (0.66-0.86)	<0.001*
SBP ≥ 120 mmHg or DBP ≥ 80 mmHg (n= 30,838)						
Nonuser	4,493/6,245 (71.95)	19,208/24,593 (78.1)	1		1	
Short-term user	960/6,245 (15.37)	2,750/24,593 (11.18)	1.49 (1.38-1.62)	<0.001*	0.70 (0.66-0.75)	<0.001*
Long-term user	792/6,245 (12.68)	2,635/24,593 (10.71)	1.28 (1.18-1.40)	<0.001*	0.82 (0.77-0.89)	<0.001*
Fasting blood glucose < 100 mg/dL (n= 24,301)						
Nonuser	3,624/4,725 (76.7)	16,210/19,576 (82.81)	1		1	
Short-term user	647/4,725 (13.69)	1,887/19,576 (9.64)	1.53 (1.39-1.69)	<0.001*	0.68 (0.63-0.74)	<0.001*
Long-term user	454/4,725 (9.61)	1,479/19,576 (7.56)	1.37 (1.23-1.53)	<0.001*	0.78 (0.71-0.85)	<0.001*
Fasting blood glucose ≥ 100 mg/dL (n= 17,124)						
Nonuser	2,419/3,560 (67.95)	10,030/13,564 (73.95)	1		1	
Short-term user	577/3,560 (16.21)	1,656/13,564 (12.21)	1.44 (1.30-1.60)	<0.001*	0.72 (0.66-0.79)	<0.001*
Long-term user	564/3,560 (15.84)	1,878/13,564 (13.85)	1.25 (1.12-1.38)	<0.001*	0.84 (0.77-0.92)	<0.001*
CCI scores = 0 (n= 26,021)						
Nonuser	3,770/4,941 (76.3)	17,154/21,080 (81.38)	1		1	
Short-term user	654/4,941 (13.24)	2,063/21,080 (9.79)	1.44 (1.31-1.59)	<0.001*	0.73 (0.67-0.79)	<0.001*
Long-term user	517/4,941 (10.46)	1,863/21,080 (8.84)	1.26 (1.14-1.40)	<0.001*	0.85 (0.78-0.93)	<0.001*
CCI scores = 1 (n= 6,679)						
Nonuser	996/1,483 (67.16)	3,893/5,196 (74.92)	1		1	
Short-term user	258/1,483 (17.4)	659/5,196 (12.68)	1.53 (1.30-1.80)	<0.001*	0.67 (0.58-0.77)	<0.001*
Long-term user	229/1,483 (15.44)	644/5,196 (12.39)	1.39 (1.18-1.64)	<0.001*	0.72 (0.62-0.83)	<0.001*
CCI scores ≥ 2 (n= 8,725)						
Nonuser	1,277/1,861 (68.62)	5,193/6,864 (75.66)	1		1	

Short-term user	312/1,861 (16.77)	821/6,864 (11.96)	1.55 (1.34-1.79)	<0.001*	0.67 (0.59-0.76)	<0.001*
Long-term user	272/1,861 (14.62)	850/6,864 (12.38)	1.30 (1.12-1.51)	<0.001*	0.80 (0.71-0.91)	<0.001*
Non diabetes history (n= 26,042)						
Nonuser	3,734/4,716 (79.18)	18,067/21,326 (84.72)	1		1	
Short-term user	579/4,716 (12.28)	1,810/21,326 (8.49)	1.55 (1.40-1.71)	<0.001*	0.66 (0.61-0.71)	<0.001*
Long-term user	403/4,716 (8.55)	1,449/21,326 (6.79)	1.35 (1.20-1.51)	<0.001*	0.76 (0.69-0.83)	<0.001*
Diabetes history (n= 15,383)						
Nonuser	2,309/3,569 (64.7)	8,173/11,814 (69.18)	1		1	
Short-term user	645/3,569 (18.07)	1,733/11,814 (14.67)	1.32 (1.19-1.46)	<0.001*	0.75 (0.68-0.82)	<0.001*
Long-term user	615/3,569 (17.23)	1,908/11,814 (16.15)	1.14 (1.03-1.26)	0.011*	0.85 (0.78-0.93)	<0.001*

Abbreviations: CCI, Charlson Comorbidity Index; SBP, Systolic blood pressure; DBP, Diastolic blood pressure; * Significance at P < 0.05. † Adjusted for age, sex, income, region of residence, SBP, DBP, fasting blood glucose, obesity, smoking, alcohol consumption, diabetes history, and CCI scores.

Table S3. Crude and overlap propensity score weighted odd ratios of dates of hydrophilic statin prescription for psoriasis.

Characteristics	N of Psoriasis	N of Control	Odd ratios for Psoriasis (95% Confidence Interval)			
	(Exposure/Total, %)	(Exposure/Total, %)	Crude	*p*-Value	Overlap Weighted Model †	*p*-Value
Age < 65 years old (n= 24,285)						
Nonuser	4,566/4,857 (94.01)	18,518/19,428 (95.32)	1		1	
Short-term user	183/4,857 (3.77)	572/19,428 (2.94)	1.30 (1.10-1.54)	0.003*	0.81 (0.70-0.93)	0.003*
Long-term user	108/4,857 (2.22)	338/19,428 (1.74)	1.30 (1.04-1.61)	0.02*	0.83 (0.70-1.00)	0.049*
Age ≥ 65 years old (n= 17,140)						
Nonuser	3,012/3,428 (87.86)	12,419/13,712 (90.57)	1		1	
Short-term user	226/3,428 (6.59)	681/13,712 (4.97)	1.37 (1.17-1.60)	<0.001*	0.76 (0.67-0.87)	<0.001*
Long-term user	190/3,428 (5.54)	612/13,712 (4.46)	1.28 (1.08-1.51)	0.004*	0.84 (0.74-0.97)	0.015*
Male (n= 23,245)						
Nonuser	4,238/4,649 (91.16)	17,421/18,596 (93.68)	1		1	
Short-term user	228/4,649 (4.9)	632/18,596 (3.4)	1.48 (1.27-1.73)	<0.001*	0.71 (0.62-0.80)	<0.001*
Long-term user	183/4,649 (3.94)	543/18,596 (2.92)	1.39 (1.17-1.64)	<0.001*	0.78 (0.68-0.90)	<0.001*
Female (n= 18,180)						
Nonuser	3,340/3,636 (91.86)	13,516/14,544 (92.93)	1		1	
Short-term user	181/3,636 (4.98)	621/14,544 (4.27)	1.18 (1.00-1.40)	0.057	0.88 (0.76-1.01)	0.062
Long-term user	115/3,636 (3.16)	407/14,544 (2.8)	1.14 (0.93-1.41)	0.212	0.92 (0.78-1.10)	0.366
Low income groups (n= 19,045)						
Nonuser	3,487/3,809 (91.55)	14,259/15,236 (93.59)	1		1	
Short-term user	182/3,809 (4.78)	567/15,236 (3.72)	1.31 (1.11-1.56)	0.002*	0.79 (0.69-0.92)	0.001*
Long-term user	140/3,809 (3.68)	410/15,236 (2.69)	1.40 (1.15-1.70)	<0.001*	0.76 (0.65-0.90)	0.001*
High income groups (n= 22,380)						
Nonuser	4,091/4,476 (91.4)	16,678/17,904 (93.15)	1		1	
Short-term user	227/4,476 (5.07)	686/17,904 (3.83)	1.35 (1.16-1.57)	<0.001*	0.77 (0.68-0.88)	<0.001*
Long-term user	158/4,476 (3.53)	540/17,904 (3.02)	1.19 (1.00-1.43)	0.056	0.91 (0.78-1.05)	0.197
Urban residents (n= 17,225)						
Nonuser	3,150/3,445 (91.44)	12,824/13,780 (93.06)	1		1	
Short-term user	161/3,445 (4.67)	550/13,780 (3.99)	1.19 (1.00-1.43)	0.056	0.88 (0.76-1.02)	0.084
Long-term user	134/3,445 (3.89)	406/13,780 (2.95)	1.34 (1.10-1.64)	0.004*	0.79 (0.67-0.94)	0.006*
Rural residents (n= 24,200)						
Nonuser	4,428/4,840 (91.49)	18,113/19,360 (93.56)	1		1	
Short-term user	248/4,840 (5.12)	703/19,360 (3.63)	1.44 (1.24-1.67)	<0.001*	0.72 (0.64-0.82)	<0.001*

Long-term user	164/4,840 (3.39)	544/19,360 (2.81)	1.23 (1.03-1.47)	0.021*	0.87 (0.76-1.01)	0.071
Underweight (n= 814)						
Nonuser	121/133 (90.98)	658/681 (96.62)	1		1	
Short-term user	9/133 (6.77)	14/681 (2.06)	3.50 (1.48-8.26)	0.004*	0.29 (0.13-0.65)	0.002*
Long-term user	3/133 (2.26)	9/681 (1.32)	1.81 (0.48-6.79)	0.377	0.57 (0.19-1.71)	0.318
Normal weight (n= 13,596)						
Nonuser	2,293/2,478 (92.53)	10,564/11,118 (95.02)	1		1	
Short-term user	117/2,478 (4.72)	333/11,118 (3.0)	1.62 (1.30-2.01)	<0.001*	0.65 (0.54-0.77)	<0.001*
Long-term user	68/2,478 (2.74)	221/11,118 (1.99)	1.42 (1.08-1.87)	0.013*	0.77 (0.62-0.97)	0.024*
Overweight (n= 11,503)						
Nonuser	2,023/2,201 (91.91)	8,667/9,302 (93.17)	1		1	
Short-term user	97/2,201 (4.41)	388/9,302 (4.17)	1.07 (0.85-1.34)	0.555	0.95 (0.79-1.14)	0.594
Long-term user	81/2,201 (3.68)	247/9,302 (2.66)	1.41 (1.09-1.81)	0.009*	0.72 (0.58-0.89)	0.003*
Obese (n= 15,512)						
Nonuser	3,141/3,473 (90.44)	11,048/12,039 (91.77)	1		1	
Short-term user	186/3,473 (5.36)	518/12,039 (4.3)	1.26 (1.06-1.50)	0.008*	0.80 (0.69-0.93)	0.004*
Long-term user	146/3,473 (4.2)	473/12,039 (3.93)	1.09 (0.90-1.31)	0.396	0.94 (0.80-1.10)	0.432
Non-smoker (n= 27,157)						
Nonuser	4,783/5,230 (91.45)	20,480/21,927 (93.4)	1		1	
Short-term user	267/5,230 (5.11)	851/21,927 (3.88)	1.34 (1.17-1.55)	<0.001*	0.78 (0.70-0.88)	<0.001*
Long-term user	180/5,230 (3.44)	596/21,927 (2.72)	1.29 (1.09-1.53)	0.003*	0.84 (0.73-0.96)	0.012*
Past and current smoker (n= 14,268)						
Nonuser	2,795/3,055 (91.49)	10,457/11,213 (93.26)	1		1	
Short-term user	142/3,055 (4.65)	402/11,213 (3.59)	1.32 (1.09-1.61)	0.005*	0.78 (0.66-0.92)	0.003*
Long-term user	118/3,055 (3.86)	354/11,213 (3.16)	1.25 (1.01-1.54)	0.042*	0.84 (0.70-1.00)	0.05
Alcohol consumption <1 time a week (n= 28,312)						
Nonuser	5,208/5,699 (91.38)	21,048/22,613 (93.08)	1		1	
Short-term user	275/5,699 (4.83)	911/22,613 (4.03)	1.22 (1.06-1.40)	0.005*	0.85 (0.76-0.96)	0.006*
Long-term user	216/5,699 (3.79)	654/22,613 (2.89)	1.33 (1.14-1.56)	<0.001*	0.80 (0.71-0.92)	0.001*
Alcohol consumption ≥1 time a week (n= 13,113)						
Nonuser	2,370/2,586 (91.65)	9,889/10,527 (93.94)	1		1	
Short-term user	134/2,586 (5.18)	342/10,527 (3.25)	1.63 (1.33-2.01)	<0.001*	0.64 (0.54-0.76)	<0.001*
Long-term user	82/2,586 (3.17)	296/10,527 (2.81)	1.16 (0.90-1.48)	0.253	0.94 (0.77-1.15)	0.533
SBP < 120 mmHg and DBP < 80 mmHg (n= 10,587)						
Nonuser	1,865/2,040 (91.42)	8,019/8,547 (93.82)	1		1	
Short-term user	96/2,040 (4.71)	304/8,547 (3.56)	1.36 (1.07-1.72)	0.011*	0.78 (0.65-0.95)	0.013*
Long-term user	79/2,040 (3.87)	224/8,547 (2.62)	1.52 (1.17-1.97)	0.002*	0.71 (0.57-0.88)	0.002*
SBP ≥ 120 mmHg or DBP ≥ 80 mmHg (n= 30,838)						
Nonuser	5,713/6,245 (91.48)	22,918/24,593 (93.19)	1		1	
Short-term user	313/6,245 (5.01)	949/24,593 (3.86)	1.32 (1.16-1.51)	<0.001*	0.78 (0.70-0.87)	<0.001*
Long-term user	219/6,245 (3.51)	726/24,593 (2.95)	1.21 (1.04-1.41)	0.015*	0.88 (0.78-1.00)	0.049*
Fasting blood glucose < 100 mg/dL (n= 24,301)						
Nonuser	4,377/4,725 (92.63)	18,539/19,576 (94.7)	1		1	
Short-term user	210/4,725 (4.44)	624/19,576 (3.19)	1.43 (1.22-1.67)	<0.001*	0.73 (0.64-0.84)	<0.001*
Long-term user	138/4,725 (2.92)	413/19,576 (2.11)	1.42 (1.16-1.72)	<0.001*	0.77 (0.65-0.90)	0.001*
Fasting blood glucose ≥ 100 mg/dL (n= 17,124)						
Nonuser	3,201/3,560 (89.92)	12,398/13,564 (91.4)	1		1	
Short-term user	199/3,560 (5.59)	629/13,564 (4.64)	1.23 (1.04-1.44)	0.015*	0.84 (0.73-0.96)	0.012*
Long-term user	160/3,560 (4.49)	537/13,564 (3.96)	1.15 (0.96-1.38)	0.12	0.91 (0.78-1.05)	0.206
CCI scores = 0 (n= 26,021)						
Nonuser	4,584/4,941 (92.77)	19,828/21,080 (94.06)	1		1	
Short-term user	206/4,941 (4.17)	710/21,080 (3.37)	1.26 (1.07-1.47)	0.005*	0.83 (0.73-0.94)	0.004*
Long-term user	151/4,941 (3.06)	542/21,080 (2.57)	1.21 (1.00-1.45)	0.046*	0.90 (0.77-1.04)	0.15

CCI scores = 1 (n= 6,679)						
Nonuser	1,329/1,483 (89.62)	4,759/5,196 (91.59)	1		1	
Short-term user	82/1,483 (5.53)	236/5,196 (4.54)	1.24 (0.96-1.61)	0.098	0.81 (0.65-1.02)	0.068
Long-term user	72/1,483 (4.86)	201/5,196 (3.87)	1.28 (0.97-1.69)	0.077	0.80 (0.63-1.02)	0.073
CCI scores ≥ 2 (n= 8,725)						
Nonuser	1,665/1,861 (89.47)	6,350/6,864 (92.51)	1		1	
Short-term user	121/1,861 (6.5)	307/6,864 (4.47)	1.50 (1.21-1.87)	<0.001*	0.68 (0.56-0.82)	<0.001*
Long-term user	75/1,861 (4.03)	207/6,864 (3.02)	1.38 (1.06-1.81)	0.019*	0.76 (0.60-0.95)	0.017*
Non diabetes history (n= 26,042)						
Nonuser	4,412/4,716 (93.55)	20,282/21,326 (95.1)	1		1	
Short-term user	181/4,716 (3.84)	646/21,326 (3.03)	1.29 (1.09-1.52)	0.003*	0.78 (0.68-0.89)	<0.001*
Long-term user	123/4,716 (2.61)	398/21,326 (1.87)	1.42 (1.16-1.74)	<0.001*	0.72 (0.61-0.85)	<0.001*
Diabetes history (n= 15,383)						
Nonuser	3,166/3,569 (88.71)	10,655/11,814 (90.19)	1		1	
Short-term user	228/3,569 (6.39)	607/11,814 (5.14)	1.26 (1.08-1.48)	0.003*	0.78 (0.68-0.90)	<0.001*
Long-term user	175/3,569 (4.9)	552/11,814 (4.67)	1.07 (0.90-1.27)	0.467	0.93 (0.80-1.08)	0.348

Abbreviations: CCI, Charlson Comorbidity Index; SBP, Systolic blood pressure; DBP, Diastolic blood pressure; * Significance at P < 0.05. † Adjusted for age, sex, income, region of residence, SBP, DBP, fasting blood glucose, obesity, smoking, alcohol consumption, diabetes history, and CCI scores.
